# Optimising a vortex fluidic device for controlling chemical reactivity and selectivity

**DOI:** 10.1038/srep02282

**Published:** 2013-07-25

**Authors:** Lyzu Yasmin, Xianjue Chen, Keith A. Stubbs, Colin L. Raston

**Affiliations:** 1 Centre for Strategic Nano-Fabrication, School of Chemistry and Biochemistry, The University of Western Australia, 35 Stirling Hwy, Crawley, W.A. 6009, Australia; 2 School of Chemistry and Biochemistry, The University of Western Australia, 35 Stirling Hwy, Crawley, W.A. 6009, Australia; 3 School of Chemical and Physical Sciences, Flinders University, Bedford Park, S.A. 5042, Australia

## Abstract

A vortex fluidic device (VFD) involving a rapidly rotating tube open at one end forms dynamic thin films at high rotational speed for finite sub-millilitre volumes of liquid, with shear within the films depending on the speed and orientation of the tube. Continuous flow operation of the VFD where jet feeds of solutions are directed to the closed end of the tube provide additional tuneable shear from the viscous drag as the liquid whirls along the tube. The versatility of this simple, low cost microfluidic device, which can operate under confined mode or continuous flow is demonstrated in accelerating organic reactions, for model Diels-Alder dimerization of cyclopentadienes, and sequential aldol and Michael addition reactions, in accessing unusual 2,4,6-triarylpyridines. Residence times are controllable for continuous flow processing with the viscous drag dominating the shear for flow rates >0.1 mL/min in a 10 mm diameter tube rotating at >2000 rpm.

Manipulating fluids in micrometre dimensions is central to microfluidic lab-on-a-chip devices which have a plethora of applications ranging from microanalysis, molecular biology, microelectronics, and to new territories in chemistry and biochemistry^1^,^2^. Microfluidics also encompasses dynamic fluidic thin films generated centrifugally by passing liquids over rotating surfaces, as in spinning disc processors (SDPs)^3^,^4^,^5^,^6^,^7^,^8^,^9^,^10^,^11^,^12^,^13^,^14^,^15^,^16^,^17^,^18^ and horizontally aligned rotating tube processors (RTPs) with open ends^18^,^19^,^20^,^21^,^22^,^23^,^24^. These processors are effective in controlling chemical reactions^3^,^4^,^5^,^6^,^7^,^8^,^9^,^10^,^11^,^12^,^13^,^22^, probing the structure of self organised systems^14^, and exfoliation and scrolling of graphite and hexagonal boron nitride (*h*-BN)^3^,^18^,^19^,^20^,^21^,^22^,^23^,^24^. However, the volumes of liquid required for maintaining constant shear intensity in the fluids flowing over the rotating surfaces, coupled with finite residence times and high cost of construction, can limit the scope of their applications. Herein we report the details of a low cost vortex fluidic device (VFD) which has a rapidly rotating tube open at one end, where at high rotational speed, intense shear is generated in the resulting thin films for finite sub-millilitre volumes of liquid, depending on the speed and orientation of the tube, and other operating parameters. This is the ‘confined mode' of operation of the VFD. It can also operate under the ‘continuous flow mode' with jet feeds delivering liquid into the rotating tube where additional shear is generated in the thin films from the viscous drag as the liquid whirls along the tube. We have recently established that the VFD is effective in exfoliating graphite and *h*-BN^25^,^26^, controlling the decoration of palladium nano-particles on carbon nano-onions arising from the high mass transfer of hydrogen gas^27^ and also palladium nano-particles on graphene^28^, disassembling self organised molecular capsules^14^,^29^, the synthesis of superparamagnetic magnetite nanoparticles embedded in polyvinylpyrrolidone followed by entrapping microalgal cells within this material, and similarly in entrapping them in graphene oxide^30^,^31^. The details of the VFD and the different types of shear regimes are now reported using selected organic reactions, initially for Diels-Alder dimerization of cyclopentadienes in optimising the operational parameters of the VFD, then sequential aldol condensation and Michael addition reactions, in gaining direct access to unusual 2,4,6-triarylpyridines which are not possible or inherently difficult and/or of limited practical convenience using traditional batch processing. Overall, the results establish dramatic control of reactivity and selectivity using the VFD, which also further highlights the versatility of the device.

Continuous flow devices can facilitate translation of small scale research production to industrial production, minimise waste and energy usage, and can be inherently safer^32^. These devices include SDP and RTP which overcome the anisotropic thermal environment, and the poor mass and heat transfer observed using traditional batch (flask) processing which can lead to mixtures of products^9^. The waves and ripples generated on the surface of dynamic thin films in SDP and RTP break down surface tension resulting in high mass transfer^14^,^15^, and the thinness of the films ensures rapid heat transfer between the liquid and rotating surface^10^,^16^. SDP has feed jets directed close to the centre of a rapidly rotating disc where there is intense micro-mixing, with residence times of typically less than a second, depending on the size of the disc, spinning speed, surface texture of the disc itself, viscosity of liquid and flow rates^10^. With respect to applications in organic synthesis, the SDP has been used to isomerize α-pinene^7^, control polymerization reactions^3^,^6^,^8^, and prepare 1,5-diketones as an entry to 2,4,6-triaryl pyridines^9^. Intriguingly, for the latter, several passes are required to move beyond the chalcone intermediate to form significant quantities of the desired 1,5-diketone^9^. The RTP has jet feeds delivering solutions intensely mixed at one end to go through the rapidly rotating tube, with the product collected at the other end^18^,^19^,^20^,^21^,^22^,^23^,^24^. Here the residence times can be minutes, depending on flow rates and the length of the tube.

To overcome limitations in these devices and introduce more flexibility in parameters, the new vortex fluidic device (VFD) was designed to operate in the same horizontal position as the RTP, as well as being able to operate at different angles, defined by the tilt angle, θ, with a closed end of the tube and jet feeds for delivering liquids and any gases from the other end, which is where the resulting processed liquid departs under continuous flow operation (Figure 1a). Shear in the thin films formed in the VFD arise from the viscous drag as for the SDP and RTP, as well as a contribution from a combination of centrifugal force with gravity, for operating at tilt angles >0°, as an additional advantage beyond the SDP and RTP, along with the ability to use smaller volumes of liquid, and a much lower cost of construction. Another advantage of VFD is that it can also operate in a so-called confined mode for a finite volume of liquid, for extending the shearing time, where the instability of the thin films arises exclusively from a combination of centrifugal force and gravity^25^.

## Results

The prototype of the VFD presented herein operates with a 10 mm diameter tube, for speeds up to 10,000 rpm and temperatures up to 200°C. Overall the device has several reproducible operating parameters including rotating speed, tilt angle θ, and flow rates, along with operating parameters for traditional batch operated reactions, such as varying the temperature and concentration of reactants.

**Figure 1:**
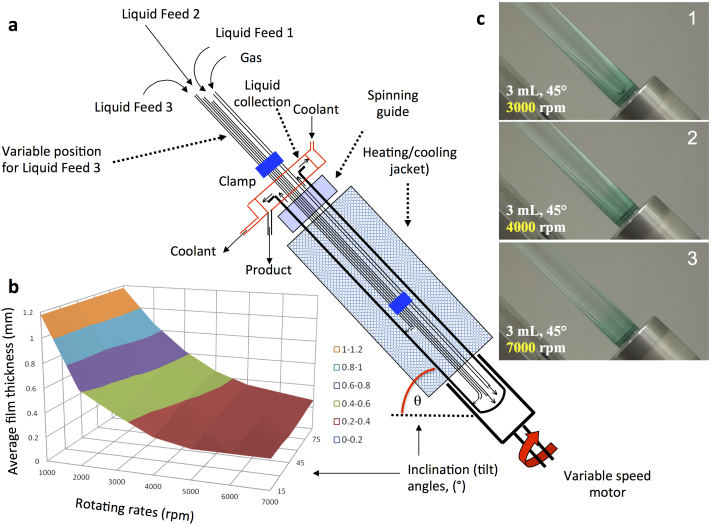
Vortex fluidic device (VFD). (a) Cross section showing the components of the device. (b) Average film thickness (mm) versus tilt angle θ, which was determined mathematically from the surface coverage of the film for 1 mL of water containing a low concentration of dye, in a standard 10 mm NMR tube with distances along its length marked externally to establish the upper level of the liquid. (c) Photographs showing the film of liquid developed for different speeds, for 3 mL of an aqueous solution, for θ = 45°, with 3000 rpm showing a quasi-parabolic film.

To gain insight into the average film thickness for steady state conditions, we determined the height of the thin film in the tube, and thus the internal area of liquid coverage, for a fixed volume of liquid (Figure 1b). For example, the average thickness of the liquid film for 1 mL of water in the 10 mm tube inclined at 45° and rotating speed at 7000 rpm is ~230 μm. We found that the shape of the film is quasi-parabolic and where the vortex extends to the base of the tube for high rotating speeds, it approximates to a uniform film (Figure 1c). On the other hand if the liquid does not form a complete vortex to the bottom of the tube, the fluid flow becomes more complex.

**Figure 2:**
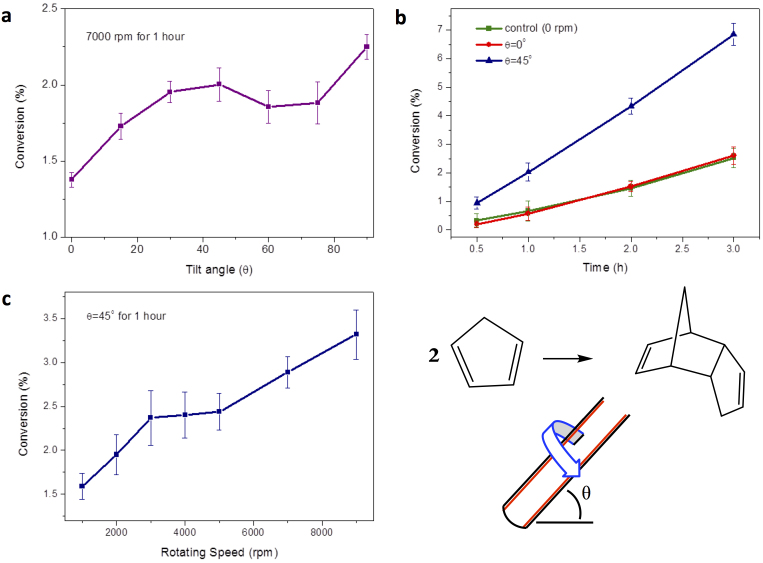
Confined mode dimerization of cyclopentadiene (CpH). Relative percent dimerization of (a) CpH versus tilt angle θ at 7000 rpm for 1 hour, (b) CpH versus time at 7000 rpm, and (c) CpH versus speed at θ = 45° for 1 hour.

**Figure 3:**
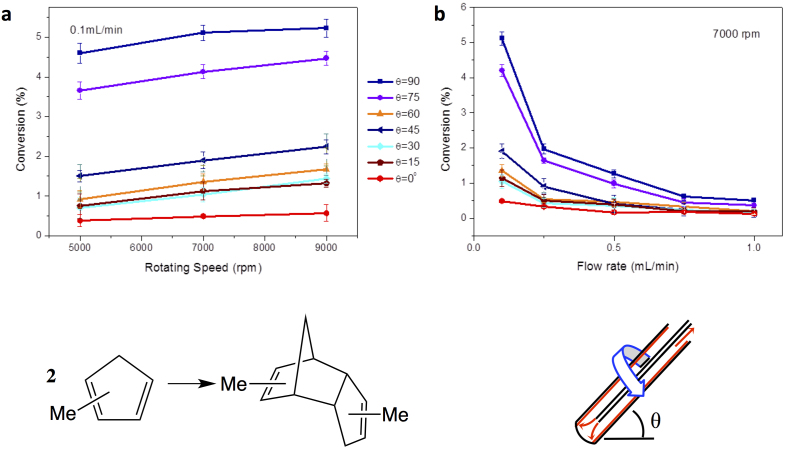
Continuous flow dimerization of methylcyclopentadiene (MeCpH). Relative percent dimerization of (a) MeCpH at various speeds for different θ values, at a flow rate of 0.1 mL/min, and (b) MeCpH with varying flow rate at different θ values at 7000 rpm, for a single pass through the VFD.

To understand the effect of the VFD on molecular reactions, we investigated the dimerization of neat cyclopentadiene and methylcyclopentadiene for different rotating speeds and tilt angles, at room temperature, under confined mode (Figure 2) and continuous flow mode (Figure 3), respectively. In confined mode, fixing the speed at 7000 rpm, the processing time at 1 hour and varying the tilt angle for 0.2 mL of cyclopentadiene results in an increase in percent conversion to the dimer, for θ > 0 (Figure 2a). As θ increases the percent dimerization increases to a plateau at ca 45° then decreases before increasing again as θ reaches 90°. Given the practical inconvenience of operating at this angle, especially when operating under continuous flow, the optimum shear for confined mode was set at θ = 45°, which corresponds to the optimum angle for exfoliation of graphite and *h*-BN, and also for continuous flow dimerization conditions at low flow rates (see below)^25^. For 7000 rpm and θ = 45°, for the same volume of liquid, there is an increase in percent dimerization over time relative to the static control and for θ = 0° (Figure 2b). At θ = 0° the liquid will rotate close to the same speed as the tube, with no shear, and consistent with this is that the outcome is the same as the static control sample, with 2.5% conversion after 3 hours, compared with 6.8% conversion in the VFD. For comparison, agitation using conventional rapid magnetic stirring results in some additional dimerization, increasing from 2.5% to 3.6% conversion for the same period. For θ = 45°, the percent dimerization increases for increasing speed, for 1 hour processing, from ca 2000 rpm to 10,000 rpm (Figure 2c). Operating speeds ≥2000 rpm under this confined mode ensures that the resulting vortex is maintained to the base of the tube, for a more uniform shear present in the thin film, which is consistent with our initial observations (Figure 1b).

To investigate VFD under continuous flow we used methylcyclopentadiene to overcome the evaporative loss of cyclopentadiene at room temperature, and found that the percent dimerization increases dramatically relative to the confined mode. For example, 0.2 mL of methylcyclopentadiene at room temperature for 1 hour with θ = 45° for the tube rotating at 7000 rpm results in 0.75% dimerization (compared with 0.34% for the static control) whereas for a single pass under continuous flow conditions for the 15 cm long tube, with θ = 45° for a flow rate of 0.1 mL/min, there is 1.9% dimerization, for which the residence time on the tube is approximately 12 minutes. For a low constant flow rate of 0.1 mL/min with the tube rotating at 7000 rpm, the percent dimerization increases for increasing speed, although this is small for θ = 0° (Figure 3a). As θ increases the dimerization increases up to 45°, then decreases up to 60°, followed by progressive increases at 75 and 90°, which is consistent with the variation in dimerization observed in the confined mode.

Dimerization of methylcyclopentadiene under different flow rates, with the tube rotating at 7000 rpm, varies dramatically for lower flow rates (Figure 3b). At the maximum flow rate (1.0 mL/min) there is essentially no difference in the percent conversion with variation in θ, but significant differences develop in tracking to the lowest flow rate (0.1 mL/min) where the conversion increases as θ increases to 45°, decreases as it approaches 60° then increases again towards 90°. For θ = 45° there is a four-fold increase in dimerization relative to θ = 0°, and effectively establishes the contribution from the shear associated with viscous drag as the liquid moves along the horizontal tube (θ = 0°), and the additional contribution to shear established in the confined mode (see above) for θ > 0°. At low flow rates the film thickness is expected to be smaller than at high flow rates for the same speed, and with thicker films the overall contribution of the shear from the viscous drag will be reduced. The increase in percent conversion at high θ (≥75°) then establishes a different shear regime, albeit of less practical convenience where the mass of the liquid has to move up the side of the tube against gravity. The low conversions observed at low speeds for higher θ values relates to these thicker films and the resultant less shear as the liquid moves along and up the tube in overcoming the effect of gravity.

**Figure 4:**
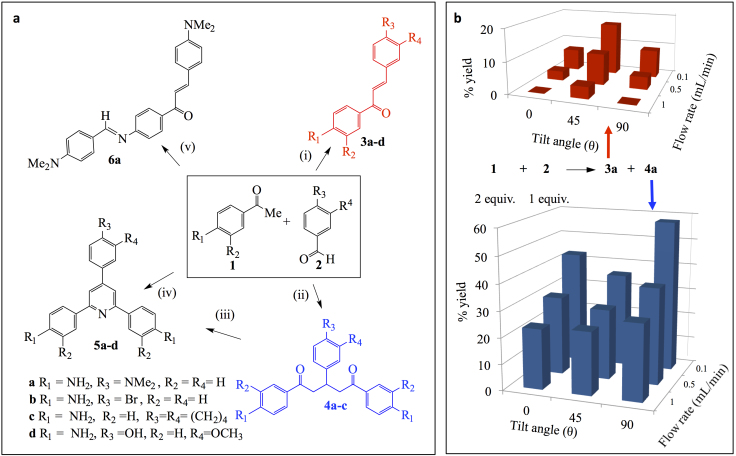
Synthesis of 2,4,6-triarylpyridienes. (a) Reaction conditions: (i) 1:1 equivalent of ketone (**1**) and aldehyde (**2**), NaOH (1 eqv.), 80°C (or room temperature for **3b** and **3c**), PEG300, confined mode VFD, θ = 45°, 7000 rpm, 30 mins, percentage yield: 52% **3a**, 65% **3b**, 61% **3c**, 20% **3d**; (ii) 2:1 equivalent of ketone (**1**) and aldehyde (**2**), NaOH (2 eqv.), 80°C, PEG300 (or 1-propanol for **4a**), continuous flow mode VFD, θ = 0°, 7000 rpm, 0.1 mL/min, percentage yield: 43% **4a**, 48% **4b**, 45% **4c**; (iii) NH_4_OAc (excess), 100°C, PEG300, batch mode^9^; percentage yield 94% **5a**, 78% **5b**, 91% **5c**; (iv) 2:1 equivalent of ketone (**1**) and aldehyde (**2**), NH_4_OAc (excess), 80°C, PEG300, continuous flow mode VFD, θ = 45°, 7000 rpm, 0.5 mL/min, percentage yield: 38% **5a**, 21% **5b**, 19% **5c**, 45% **5d**; (v) Batch mode, 90% **6a**^9^. (b) Product distribution for a 2:1 reaction of **1** and **2**, in generating a mixture of **3a** and **4a**.

We have translated the optimised conditions of these dimerization reactions to investigate confined and continuous flow synthesis of 2,4,6-triarylpyridines (Figure 4a). These are formed via an aldol condensation reaction to give **3a–d**, followed by a Michael addition to give **4a–c** (Figure 4). In some cases there is a need to gain control over competing reactions, as in the undesirable Schiff base condensation coupled with aldol condensation (compound **6a**), which is a favoured product using batch processing in attempting to prepare the 1,5-diketone **4a** via the chalcone **3a**. This unwanted product arises from poor heat and mass transfer which is overcome using SDP, but requires multiple passes because of the short residence time (<1 second for a 10 cm disc)^9^. A single pass, under continuous flow conditions, on the VFD now allows direct access to **4a**, albeit with low amounts of chalcone formation, depending on the choice of control parameters (Figure 4b). At low flow rate (0.1 mL/min) the percent yield is higher than at corresponding high flow rates (1 mL/min) which is expected due to the longer residence time, but high flow rate reduces the relative amount of chalcone being formed. The amount of chalcone is minimal for θ = 0 and 90°, whereas at 45° there is a larger amount of the compound, especially at a low flow rate. This again reflects a different shear regime at this angle, consistent with what has been identified in the dimerization of methyl cyclopentadiene. However, the confined mode operation of the VFD is effective in preparing the chalcones **3a–d** in high yield. Thus the choice of continuous flow versus confined mode is another parameter in gaining control over the outcome of the reactions. Interestingly the 2,4,6-triarylpyridines **5a–d** can also be formed in the VFD under continuous flow conditions in a single pass in the presence of NH_4_OAc (Figure 4a, reaction iv). This is particularly important for **5d** given that it was not possible to make the 1,5-diketone precursor, **4d**, using methods (ii) and (iii) (Figure 4a). 2,4,6-Triarylpyridines are accessible using SDP but the short residence time necessitates several passes through the microfluidic device, with less control over the product distribution^9^. They are also accessible in a one step process in the presence of NH_4_OAc using microwave irradiation^33^, but this is not immediately scalable processing, and the energy usage using microwave heating is questionable^34^.

## Discussion

The VFD has different shear regimes in the thin films. For the confined mode of operation, the shear depends on the tilt angle θ of the rapidly rotating tube, which is greatest at 45° and 90°, and the speed of the tube. This was established by the increase in dimerization of cyclopentadiene relative to controls (θ = 0°, no agitation or stirring of the liquid). For the continuous flow mode, the viscous drag dominates the shear for flow rates at 1.0 mL/min, and for flow rates approaching 0.1 mL/min the different shear established for the confined mode also become important, which has been established by the increase in dimerization of methylcyclopentadiene relative to the same controls.

The increase in shear, as judged by the increase in dimerization of the cyclopentadienes, for θ up to 45°, then a decrease before an increase as θ approaches 90°, defines different shear regimes corresponding to different orientations of the centrifugal force relative to gravity, for which the fluid dynamics is presumably complex. The findings provide a basis for understanding the outcome of recent applications of the VFD operating under confined mode, where the optimum tilt angle approximated to 45°. This includes the ‘top down' exfoliation of laminar material^25^,^26^, the ‘bottom up' growth of nano-particles^27^,^28^, disassembly of self organised systems^14^,^29^, and entrapping microalgal cells^30^,^31^. The results for the continuous flow synthesis are consistent with the synthesis of superparamagnetic nanoparticles for θ at 0° for a flow rate of 1 mL/min^30^, noting that the tilt angle under continuous flow becomes important for much slower flow rates, especially approaching 0.1 mL/min.

The shear in the confined and continuous flow modes is effective in enhancing the chemical reactions, in providing a ‘soft' form of energy to increase molecular collisions for reactions under diffusion control. The increase in percent conversion of the cyclopentadienes is possible without heating the samples, and for the formation of the 2,4,6-triarylpyridines the ability to circumvent the formation of the Schiff base, compound **6a**, establishes the ability to gain control over kinetic verses thermodynamic product. Intense micromixing and associated shear here also allows direct access to amine substituted chalcones and 1,5-diketones, without the need to prepare the corresponding nitro-compounds^35^, for then reduction of the functional groups post-aldol and Michael addition reactions, and also formation of the 2,4,6-trialylpyridine.

The above cyclopentadiene Diels-Alder reactions and the ability to use the VFD to control reactivity and selectivity in the formation of 2,4,6-triarylpyridines, establishes the utility of using the inexpensive microfluidic device for improving the outcome of chemical reactions. This coupled with the ability to scale down to sub-millilitre volumes in the confined mode or scale up under continuous flow mode while maintaining intense shear allows for controlled chemical reaction conditions to be achieved. There is potential for application of the VFD in a myriad of functional group transformations, with hierarchical control, including reactions of gases with liquids, in taking advantage of the high mass transfer of gases at the dynamic interface between the two states. A potential limitation of the VFD is continuous flow operation for highly viscous liquids and/or where the product gelates which may clog the outlet system.

The VFD is modular and can be readily modified, in changing the nature of the tube, with surface texture features for increasing the intensity of micro-mixing, and covalent attachment, in controlling the contact angle of the surface (hydrophobic/hydrophilic balance) to change the viscous drag and thus the shear intensity. The VFD can also incorporate field effects, including standard light sources and lasers, and has high throughput processing capabilities for developing libraries of nanomaterials and organic compounds. Several reactions can potentially be telescoped within a single platform, or sequential platforms, with the ability to carry out reactions in an inert atmosphere using a gas feed or by placing the small unit in a controlled atmosphere glove box, and properties of liquids under intense shear can also be studied for small volumes.

## Methods

^1^H and ^13^C NMR spectra were recorded on a Varian NMR spectrometer at 400 and 100 MHz respectively. The VFD was constructed at The University of Western Australia, and all experiments used a 10 mm NMR tube, 15 cm in length, which was capped in the confine mode dimerization studies of 0.2 mL of freshly distilled cyclopentadiene. For dimerization studies of under continuous flow mode, freshly distilled methylcyclopentadiene was kept in an ice bath prior to use, and the liquid was collected after steady state flow from the tube. The percent conversions for both studies were determined using ^1^H NMR. For the confined mode synthesis of the chalcones (**3**), a mixture of *p*-aminoacetophenone (**1**) (1 mmol), sodium hydroxide (1 mmol) and benzaldehyde (**2**) (1 mmol) in 2 mL PEG was spun in a capped tube at 7000 rpm, 45° degree tilt angle at 80°C (for **3a** and **3d**) or ambient temperature (for **3b** and **3c**) for 30 minutes. Water (25 mL) was then added and the resulting yellow precipitate collected and dried in vacuo. For continuous flow synthesis of 1,5-diones (**4**), *p*-aminoacetophenone (**1**) (1 mmol) and hydroxide (2 mmol) in PEG 300 (1 mL) was directed through one jet feed, with the other a solution of benzaldehyde (**2**) (1 mmol) in PEG 300 (1 mL). Both solutions were then passed through the VFD using continuous flow mode; 80°C, 7000 rpm, 0 degree tilt angle and 0.1 mL/min flow rate. Water was added to the processed liquid affording a yellow precipitate as mixture of 1,5-diketone (**4**) and chalcone (**3**). Similarly excess NH_4_OAc (400 mg) was used in place of NaOH, to afford the 2,4,6-triarylpyridine (**5**) directly.

## Author Contributions

X.C. carried out fluid flow experiments, L.Y. carried out the organic reactions, K.A.S. and C.L.R. designed the organic synthesis experiments and wrote the paper, all authors reviewed the manuscript, and C.L.R. designed the micofluidic platform and coordinated the research.

## Supplementary Material

Supplementary Information Supplementary Information
