# Supplementary material for: Optimising a vortex fluidic device for controlling chemical reactivity and selectivity
Source: Sci Rep. 2013 Jul 25;3:2282. doi: 10.1038/srep02282 (PMC3722563; doi:10.1038/srep02282)

Supplementary Information

Optimising a vortex fluidic device for controlling chemical reactivity and selectivity

Lyzu Yasmin,[1] Xianjue Chen,[1] Keith A. Stubbs,[2] and Colin L. Raston[*3]*

1. Centre for Strategic Nano-Fabrication, School of Chemistry and Biochemistry, The University of Western Australia, 35 Stirling Hwy, Crawley, W.A. 6009, Australia.

2. School of Chemistry and Biochemistry, The University of Western Australia, 35 Stirling Hwy, Crawley, W.A. 6009, Australia.

3. School of Chemical and Physical Sciences, Flinders University, Bedford Park, S.A. 5042, Australia

colin.raston@flinders.edu.au

Contents:

Materials and methods — Page SI-1

Vortex fluidic device — Page SI-2

Experimental procedures — Page SI-3

References — Page SI-6

Spectra — Page SI-7

Materials and methods

All commercially obtained chemicals were used as received unless otherwise specified. Dicyclopentadiene was obtained from Fluka. Methylcyclopentadiene-dimer was obtained from Alfa-Aesar. *p*-Aminoacetophenone was purchased from Fluka. *N*,*N*-

Dimethylaminobenzaldehyde was obtained from BDH. 2-Napthaldehyde, 4-bromobenzaldehyde and vanillin were purchased from Alfa Aesar, Aldrich and Unilab respectively. Thin layer chromatography (TLC) was conducted with Silica gel 60 F254. Visualization was effected by ultraviolet light (254 nm). Melting points were measured by Electrothermal. ^{1}H and ^{13}C NMR spectra were recorded on Varian NMR spectrometer at 400 MHz and 100 MHz respectively.

Vortex fluidic device

A photograph showing a prototype VFD used in the study, with explanations, is provided in Fig. S1.

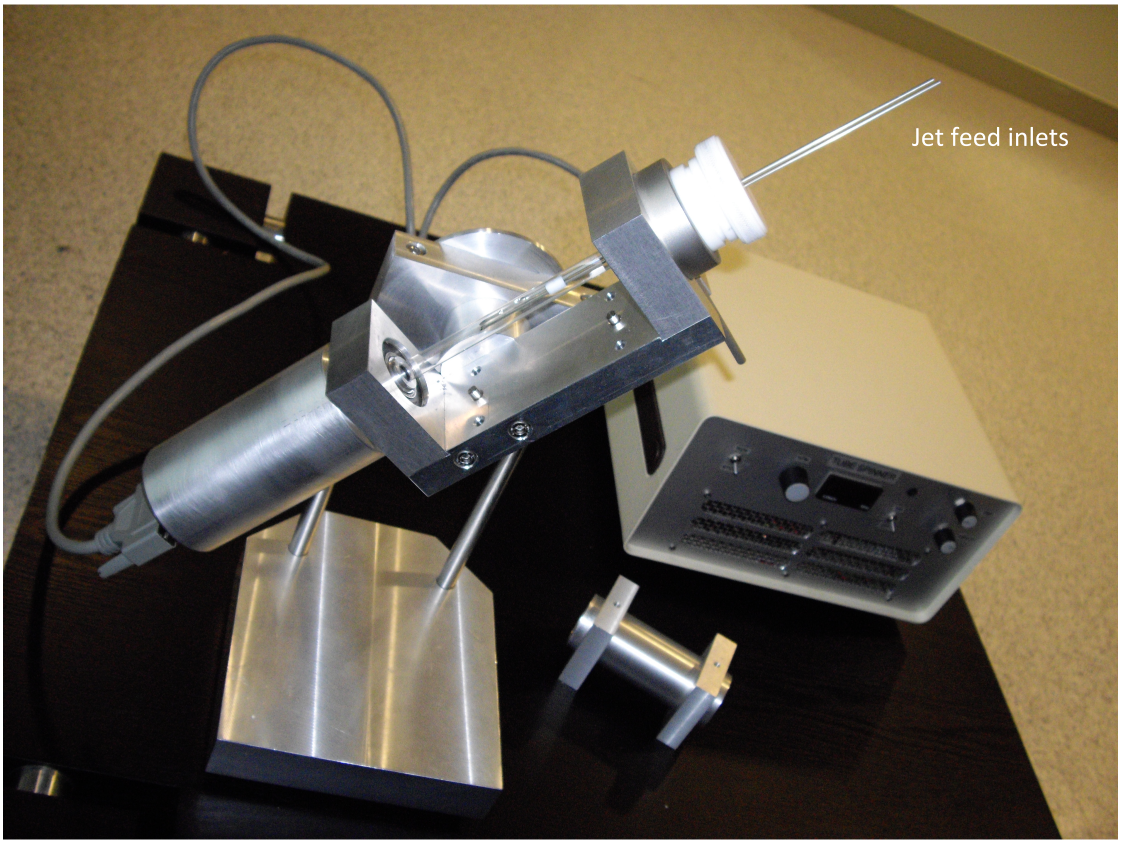

Figure S1. A prototype VFD showing the overall set up. The jet feed inlets are connected to a peristaltic pump, and the heating unit is modular, locking into place with two pins, with the tilt angle adjustable on a locking pivot system.

Experimental procedures

1. Dimerization of Cyclopentadiene and Methyl cyclopentadiene

1.1. Synthesis of Cyclopentadiene/Methyl cyclopentadiene from its Dimer:

Cyclopentadiene and methylcyclopentadiene were synthesised from their dimer by fractional distillation at 165°C and 180°C respectively before use. The monomers readily dimerize at room temperature, and thus the materials were always kept in a cool place.

1.2. Dimerization of cyclopentadiene by VFD at confined mode:

For each experiment cyclopentadiene (0.2 mL) was taken in a 10 mm diameter and 15 cm long NMR tube with a cap unless otherwise stated. Percent conversion was obtained using ^{1}H NMR analysis.

(a) Varying the angle: The speed and time of VFD were set at 7000 rpm and 1 hour respectively. Seven different experiments were carried out at seven different tilt angles (0, 15, 30, 45, 60, 75 and 90 degrees). (Fig.2-a)

(b) Varying the Time: The speed and tilt angle of VFD were set at 7000 rpm and 45° respectively. Four different experiments were conducted at four different time lengths (0.5, 1, 2 and 3 h). Similar experiments were carried out at a 0° tilt angle. (Fig.2-b)

(c) Varying the speed: The time and tilt angle of VFD were set at 1 hour and 45° respectively. The experiments were carried out at six different speeds, 1000, 2000, 3000, 5000, 7000 and 9000 rpm. (Fig.2-c)

1.3. Dimerization of methylcyclopentadiene by VFD at continuous flow mode:

For the continuous flow method, methylcyclopentadiene was kept in a sample vial which was cooled in an ice bath. Products were collected at steady state position of reactant inside the glass tube. Percent conversion was obtained using ^{1}H NMR analysis.

(a) Varying flow rates at different tilt angles: The speed was fixed at 7000 rpm for all experiments at different five flow rates (0.1, 0.25, 0.5, 0.75 and 1.0 mL/min) and at different nine tilt angles (0, 15, 30, 37.5, 45, 52.5, 60, 75 and 90 degrees). (Fig.3-a)

(b) Varying speeds at different tilt angles: The flow rate was set at 0.1mL/min for all experiments at different three speeds (5000, 7000 and 9000 rpm) and at nine different tilt angles (0, 15, 30, 37.5, 45, 52.5, 60, 75 and 90 degrees). (Fig.3-b)

2. Synthesis

2.1. General route for synthesis of chalcones (3):

p-Aminoacetophenone (**1**) (1 mmol) was added to a stirred solution of PEG 300 (1 mL) and crushed sodium hydroxide (1 mmol) in the same flask. Benzaldehyde (**2**) (1 mmol) was then added to a stirred solution of PEG 300 (1 mL) in another flask. Both solutions were then mixed in a capped 10 mm dimater glass tube and spun in the VFD at 7000 rpm, 45° degree tilt angle at 80°C (for **3a** and **3d**) or ambient temperature (for **3b** and **3c**) for 30 minutes in confined mode. Water (25 mL) was the added and the resulting yellow precipitate was collected by suction filtration and dried in vacuo.

2.2. General route for synthesis of 1,5-diones (4):

p-Aminoacetophenone (**1**) (1 mmol) and crushed sodium hydroxide (2 mmol) were added to a stirred solution of PEG 300 (1 mL) in a flask. Benzaldehyde (**2**) (1 mmol) was then added to a stirred solution of PEG 300 (1 mL) in another flask. Both solutions were then passed

through the VFD using continuous flow mode. The VFD conditions were 80°C, 7000 rpm, 0 degree tilt angle and a flow rate of 0.1mL/min. After cooling the reaction mixture to room temperature, water was added to aid precipitation. The yellow precipitate as mixture of 1,5-diketone (**4**) and chalcone (**3**) was collected by suction filtration and dried. The crude product was purified by column chromatography over silica gel using hexane:ethyl acetate (1:2) as eluent to obtain a pure product (**5**) as light brown solid.

2.3. Synthesis of 2,4,6-triarylpyridine (5) using route (iv):

p-Aminoacetophenone (**1**) (1 mmol) was added to a stirred solution of PEG 300 (1 mL) and excess NH_4OAc (400 mg) in a flask. Benzaldehyde (**2**) (1 mmol) was then added to a stirred solution of PEG 300 (1 mL) in another flask. Both solutions were passed through the VFD as a continuous flow at 80°C, 7000 rpm, 45 degree tilt angle and 0.5 mL/min flow rate. After reaction the mixture was cooled to room temperature and 50 ml water was added to afford a yellow solid product of the mixture of 2,4,6, triarylpyridine (**5**) and chalcone (**3**). The crude product was purified by column chromatography over silica gel using hexane:ethylacetate (1:2) as eluent to obtain a pure product (**5**) as brown solid.

2.4. Characterization

3c: δ_H (400 MHz, d_6-DMSO) 8.28 (s, 1H), 8.08 (dd, J=8.6 and 1.5 Hz, 1H), 7.96 (m, 6H), 7.76 (d, J=15.6 Hz, 1H), 7.56 (m, 2H), 6.64 (d, J=8.8 Hz, 2H), 6.16 (s, 2H, NH_2). δ_C (100.5 MHz, d_6-DMSO) 186.2, 154.4, 141.8, 134.1, 133.5, 133.3, 131.6, 130.3, 128.8, 128.8, 128.1, 127.5, 127.1, 125.8, 124.9, 123.2, 113.2; m.p. 175 °C.

3d: δ_H (400 MHz, d_6-DMSO) 7.89 (d, J=8.8 Hz, 2H), 7.67 (d, J=15.4 Hz, 1H), 7.53 (d, J=15.4 Hz, 1H), 7.45 (d, J=2.0 Hz, 1H), 7.19 (dd, J=2.0 and 8.2 Hz, 1H), 6.8 (d, J=8.2 Hz, 1H), 6.61 (d, J=8.8 Hz, 2H), 6.07 (s, 2H, NH_2), 3.86 (s, 3H, OCH_3). δ_C (100.5 MHz, d_6-DMSO) 186.3, 154.0, 149.4, 148.4, 131.3, 127.2, 126.1, 123.9, 119.4, 115.9, 113.1, 56.2 ; m.p. 220 °C.

4c: δ_H (400 MHz, d_6-DMSO) 7.76 (m, 4H), 7.65 (d, J= 8.8 Hz, 2H), 7.48 (dd, J= 8.6 and 1.5 Hz, 1H), 7.38 (m, 2H), 6.5 (d, J= 8.8 Hz, 4H), 5.97 (s, 4H, NH_2). δ_C (100.5 MHz, d_6-DMSO) 196.1, 154.0, 142.9, 133.4, 132.2, 131.0, 131.0, 127.8, 127.8, 127.0, 126.2, 126.1, 125.6, 125.1, 112.9, 43.9, 38.1; m.p. 188 °C.

5c: δ_H (400 MHz, d_6-DMSO) 8.54 (s, 1H), 8.0-8.1 (m, 7H), 7.96 (d, J= 8.3 Hz, 1H), 7.92 (s, 2H, Py-H), 7.55 (m, 2H), 6.68 (J= 7.9 Hz, 4H), 5.4 (s, 4H, NH_2). δ_C (100.5 MHz, d_6-DMSO) 157.1, 150.3, 148.8, 136.2, 133.7, 133.4, 128.9, 128.9, 128.2, 128.0, 127.1, 127.0, 126.9, 126.6, 125.4, 114.1, 113.3 ; m.p. 170 °C.

5d: δ_H (400 MHz, d_6-DMSO) 7.99 (d, J=8.2 Hz, 4H), 7.73 (s, 2H, Py-H), 7.43 (d, J=2 Hz, 1H), 7.37 (dd, J=2 and 8.9 Hz, 1H), 6.9 (d, J=8.2 Hz,1H), 6.67 (d, J=8.2 Hz, 4H), 5.38 (s, 4H, NH_2), 3.92 (s, 3H, OCH_3). δ_C (100.5 MHz, d_6-DMSO) 156.8, 149.1, 148.4, 148.1, 130.1, 128.1, 127.2, 120.3, 116.2, 114.0, 112.6, 111.5, 56.4; m.p. 178 °C.

The ^{1}H and ^{13}C NMR data of **3a, 3b, 4a, 4b, 5a, 5b** and **6a** were consistent with all literature values.[1-3]

References:1. Smith, N. M, Corry, B., Iyer, K. S., Norret, M. & Raston, C. L. A microfluidic platform to synthesise a G-quadruplex binding ligand. *Lab Chip* **9**, 2021-2025(2009).

2. Smith, N. M., Raston, C. L., Smith, C. B. & Sobolev, A. N. PEG mediated synthesis of amino-functionalised 2,4,6-triarylpyridines. *Green Chem*. **9**, 1185-1190(2007).

3. Smith, N. M., Pengkai, S., Nithiananthan, A., Norret, M., Stewart, G. A. & Raston, C. L. Immunomodulatory effects of functionalised chalcones on pro-inflammatory cytokine release from lung epithelial cells. *New J. Chem.* **33**, 1869-1873(2009).

^{1}H NMR spectrum of **3c**

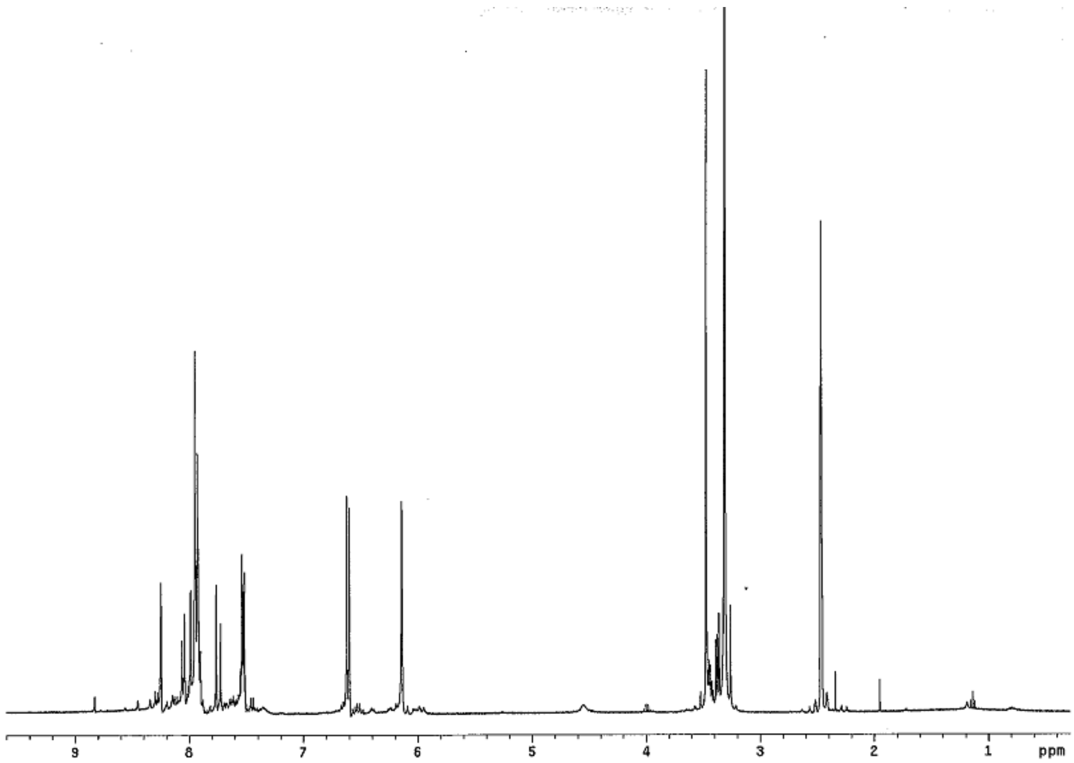

^{13}C NMR spectrum of **3c**

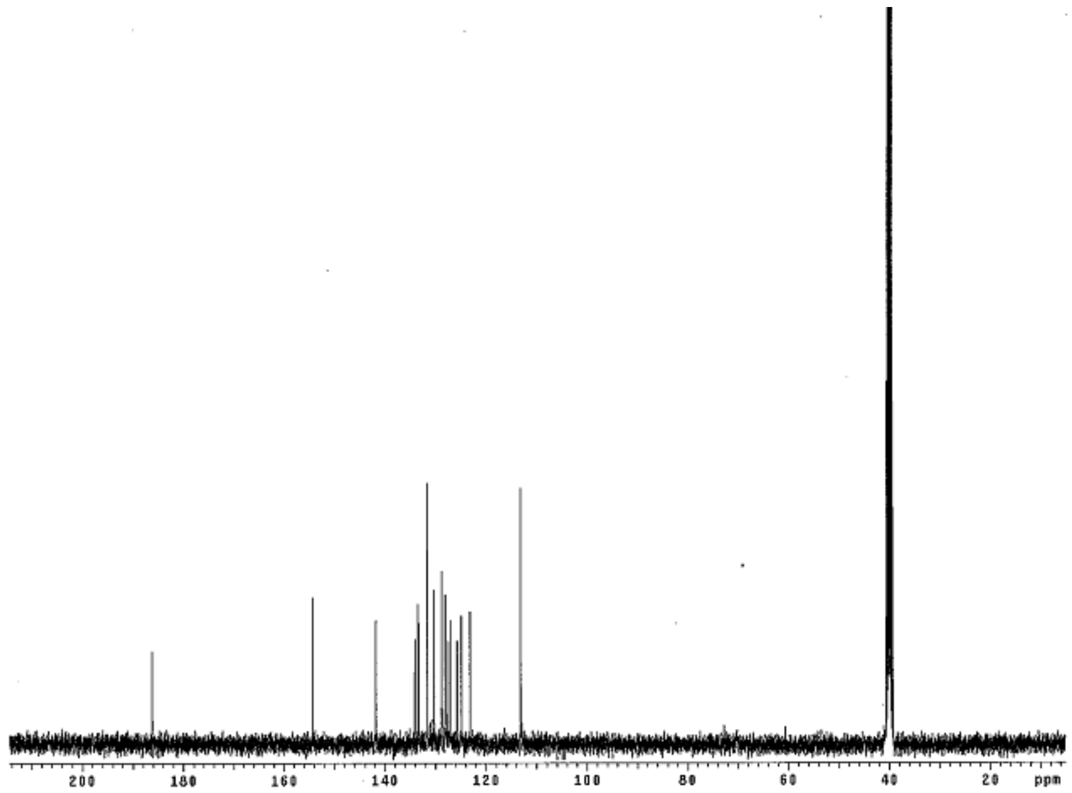

^{1}H NMR spectrum of **3d**

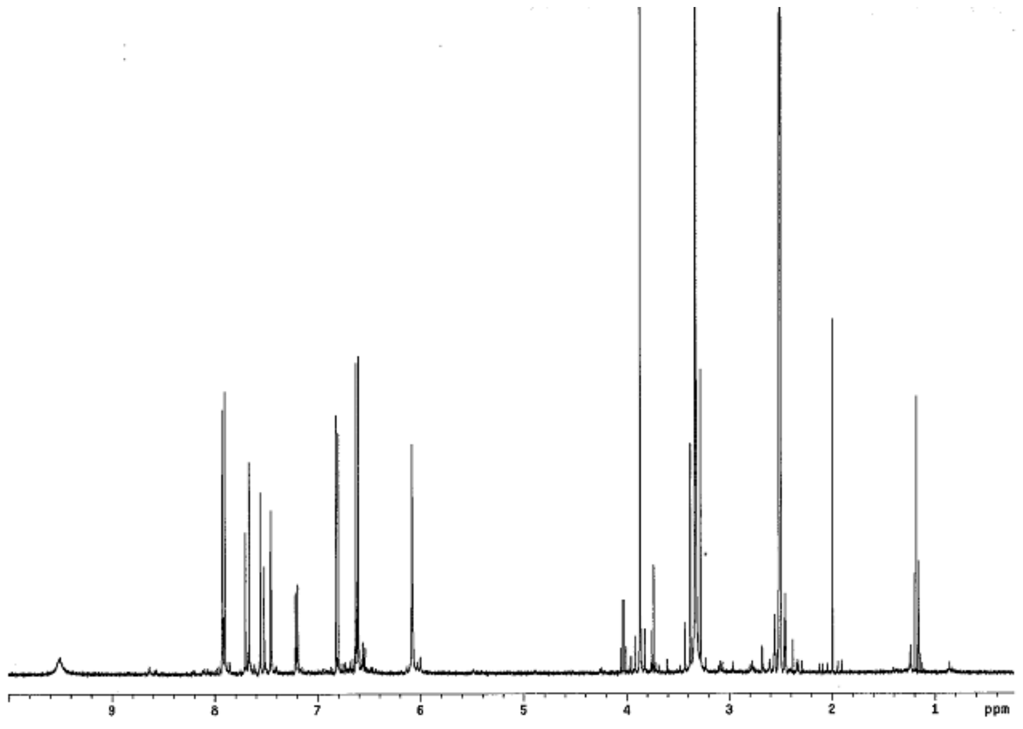

^{13}C NMR spectrum of **3d**

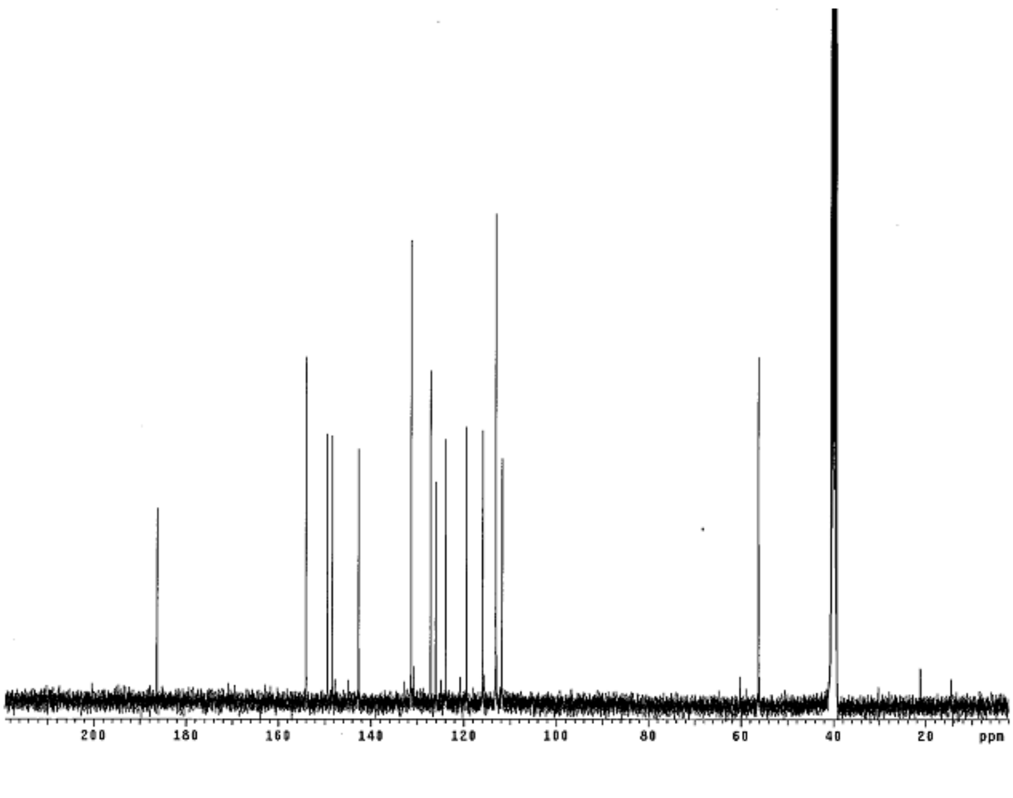

^{1}H NMR spectrum of **4c**

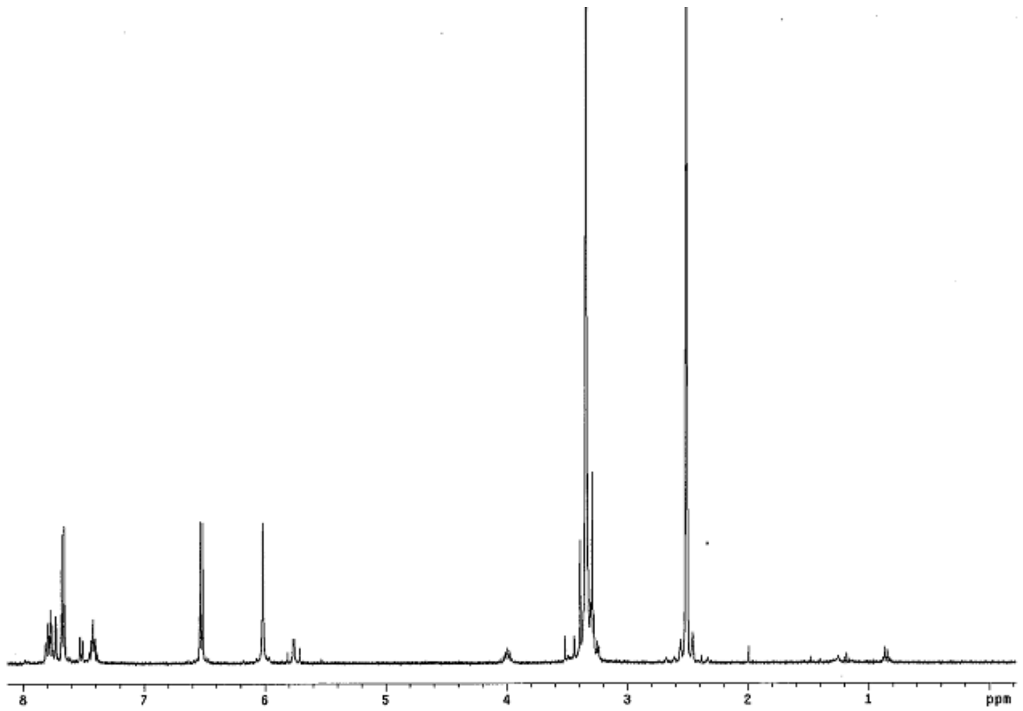

^{13}C NMR spectrum of **4c**

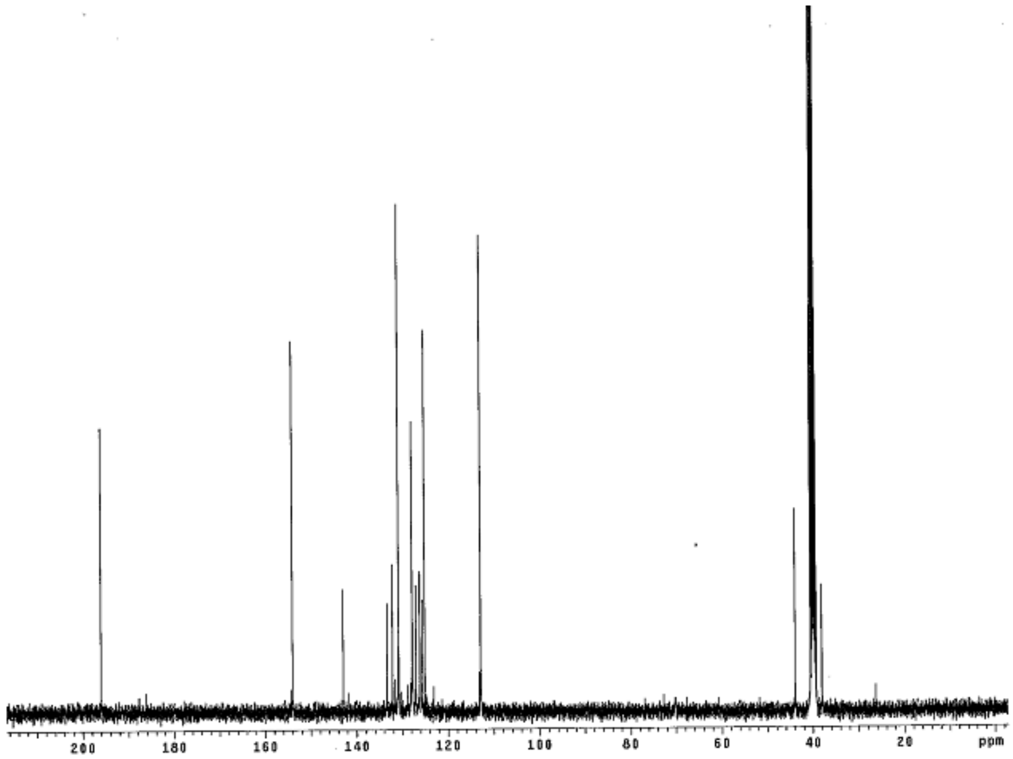

^{1}H NMR spectrum of **5c**

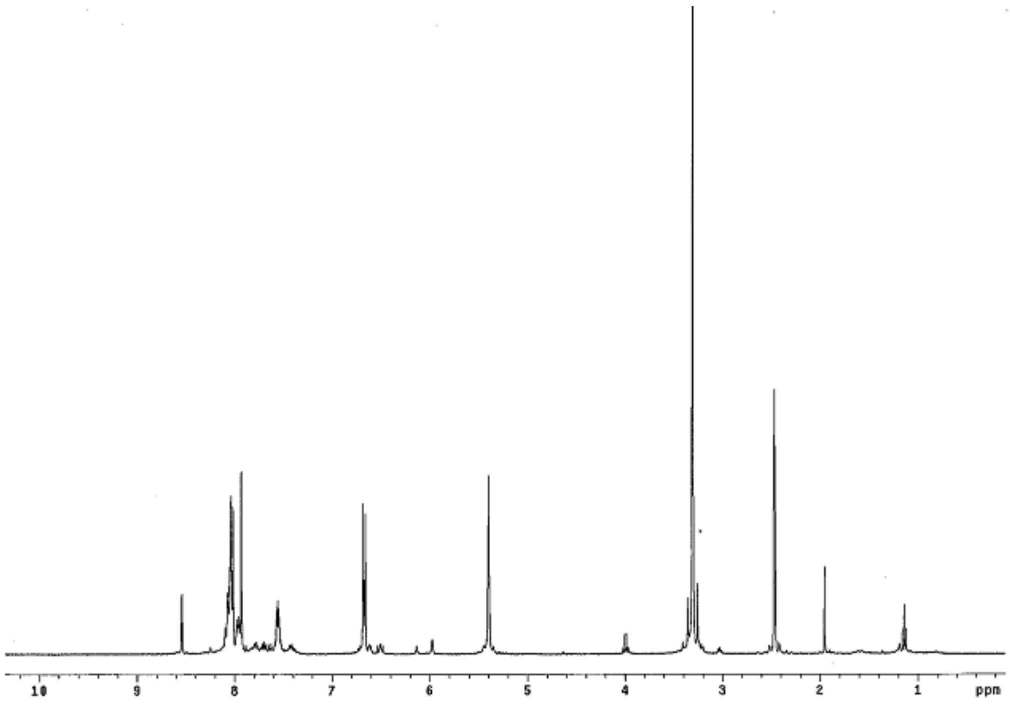

^{13}C NMR spectrum of **5c**

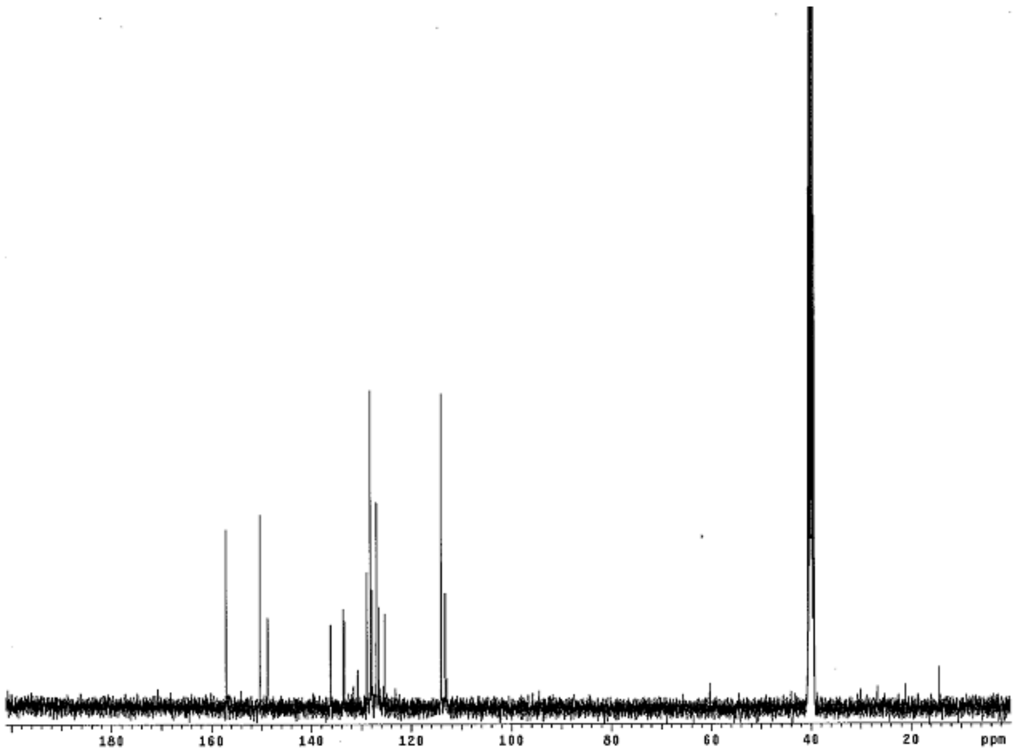

^{1}H NMR spectrum of **5d**

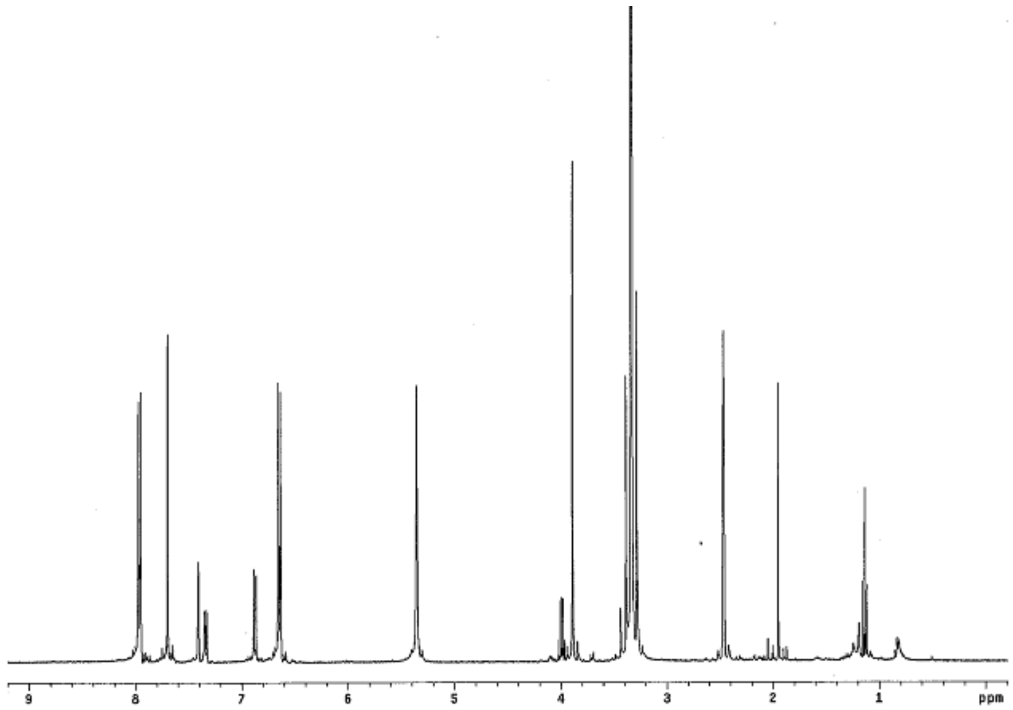

^{13}C NMR spectrum of **5d**

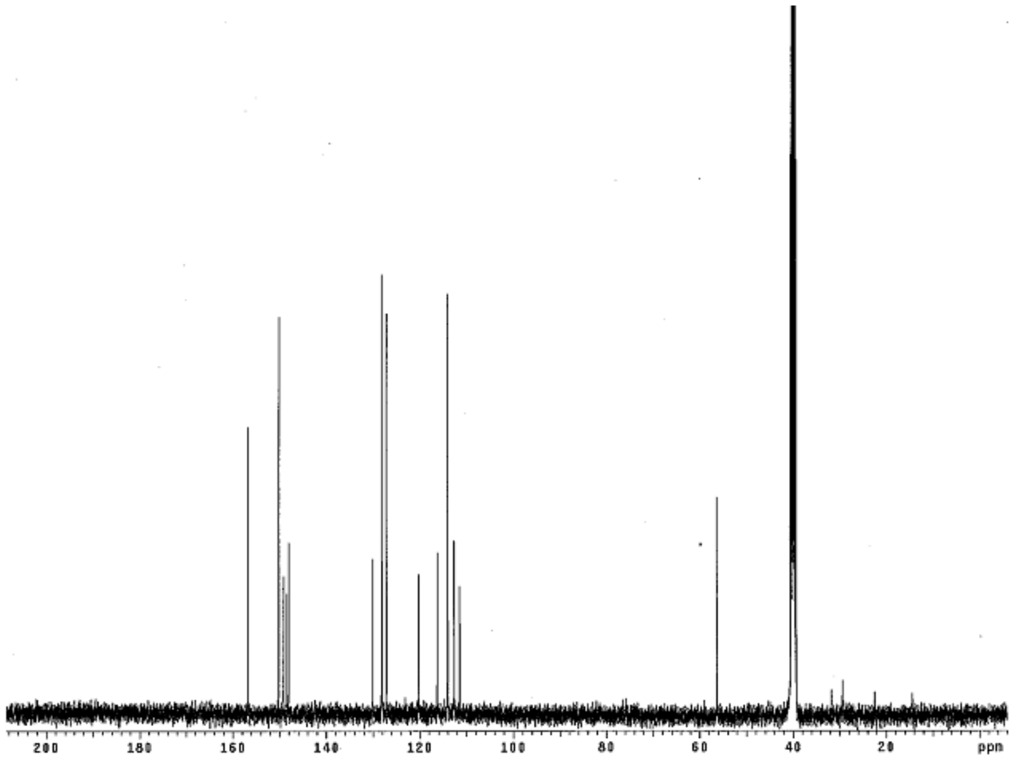

Supplement: Supplementary Information [file srep02282-s1.pdf]
